# Exaggerated envy and guilt measured by economic games in Japanese women with anorexia nervosa

**DOI:** 10.1186/s13030-018-0138-8

**Published:** 2018-12-06

**Authors:** Masanori Isobe, Michiko Kawabata, Ema Murao, Tomomi Noda, Noriko Matsukawa, Ryosaku Kawada, Teruhisa Uwatoko, Toshiya Murai, Shun’ichi Noma, Hidehiko Takahashi

**Affiliations:** 1 Department of Psychiatry, Graduate School of Medicine, Kyoto University, 54 Shogoin-Kawahara-cho, Sakyo-ku, Kyoto, 606-8507 Japan; 2 Department of Health Service, Kyoto University, Yoshidahonmachi, Kyoto, 606-8507 Japan

**Keywords:** Anorexia nervosa, Ultimatum game, Fairness, Envy, Guilt

## Abstract

**Background:**

Anorexia nervosa (AN) patients are assumed to express high levels of guilt and envy. Ultimatum game (UG) is a standard behavioral task that focuses on interpersonal behavior when splitting a sum of money between two players. UG studies consistently demonstrate that people tend to decrease their inequity in outcomes, one explanation being that economically irrational decision-making may partly arise from the emotions guilt and envy. We assumed that AN patients would perform excessively fair in UG, reflecting high guilt and envy.

**Methods:**

We utilized UG to investigate the characteristics of guilt and envy among 24 Japanese AN patients and 22 age-matched healthy controls (HC). The relation between the outcome of UG and decision strategy confirmed by post-experimental questionnaires was analyzed.

**Results:**

As proposer, AN offered a larger amount to the responder compared with HC (*p* = 0.002) while, on the other hand, as responder, AN demanded much higher allocation to accept the offer compared with HC (*p* = 0.026). Regarding the strategy as responder, AN put more emphasis on fairness and less emphasis on monetary reward compared with HC (*p* = 0.046, *p* = 0.042, respectively).

**Conclusions:**

The results indicate that Japanese AN patients demonstrate strong preference for fairness, with high guilt and high envy. High sensitivity to guilt and envy of AN patients can affect not only their own behavior concerning eating attitude and body shape, but also decision-making in interpersonal situations. Behavioral experimental settings among social situations will enable us to evaluate and help actual decision-making in the real life of patients.

## Introduction

Anorexia nervosa (AN) is characterized by distorted body image and excessive dieting [1]. Individuals with AN feel guilt for “indulgent” eating or loss of control in an eating setting [2], and they feel envy when they see someone very slim [3]. In this manner, those with AN are assumed to express high levels of guilt and envy [4, 5]. However, these emotional reactions can be observed not only in food/body contexts but also in more general situations. Adolescents with AN showed more increased guilt than healthy ones [6], and increased envy may have roots deep in the psychopathology of AN [7].

To understand these emotions, economics games, which can assess decision-making in social situations [8] and provide a quantitative value to examining the psychopathology by predicting optimal adaptation to a changing environment [9], have been used in psychiatric populations [10, 11]. In particular, ultimatum game (UG), focusing on interpersonal behavior when splitting a sum of money between two players, is one of the most widely used tasks, as guilt and envy are addressed with this task [12]. The proposer suggests the distribution amount, and then the responder decides to accept or reject the proposal. If the responder accepts, the proposed distribution is the final allocation for the two players. If the responder rejects the proposal, both players receive nothing. Although theory predicts that the responder accepts all offers and that the proposer offers the smallest amount [13], behavioral economics consistently demonstrates that people do not necessarily maximize their allocation [14]. It has been assumed that people tend to minimize inequity in outcomes [11]. One explanation for such behavior is that guilt and envy play roles in the game. Rejecting low offers in UG implies envy, which is the preference to prevent the opposing person earning more than oneself, and offering equal allocation is derived from the desire to minimize guilt of having too much [15]. Given that both guilt and envy could be measured in the two different roles of UG (proposer, responder), we decided to use UG with the aim of quantifying these emotions in social situations among AN patients. We assumed that behaviors with exaggerated envy and guilt could be detected in AN patients, resulting in a high rejection rate of low offers and egalitarian allocation.

## Method

### Participants

The AN patient group consisted of 24 Japanese female patients who were referred to the Department of Psychiatry of Kyoto University Hospital. Each patient fulfilled the criteria for AN based on the Structural Clinical Interview for DSM-IV Axis I Disorders (SCID) Patient Edition. Twelve patients were classified as the restricting subtype (ANR) and 12 as the binge-eating/purging subtype (ANBP). All the patients were also screened with the SCID Patient edition about any psychiatric comorbidity, and none were found to be comorbid with any other psychiatric disorders. Predicted premorbid IQ was measured by the Japanese Version of the National Adult Reading Test short form (JART) [16], and eating behavior was measured by Eating Disorder Examination-questionnaire 6.0 (EDE-Q). The HC group consisted of 22 Japanese healthy female individuals age-matched to the AN group. They were evaluated using the SCID Non-patient Edition, and were found to have no history of psychiatric disease. All participants of both groups were physically healthy at the time of participation, and had no history of neurological injury or disease, severe medical disease, or substance abuse.

### Ultimatum game

Participants acted as both proposer and responder in 20 trials each. The participants were told that the total sum was 1000 Japanese Yen (JPY; approximately 9 US Dollars), and that they had to divide this sum between self and the partner. All the tasks were performed by computer. First, they played the role of responder. Prior to the task, they were told that “the preceding participants had proposed the distribution in advance,” and they were asked to accept or to reject the distribution. The proposed amounts were 100, 200, 300, 400 and 500 JPY, and each offer was proposed four times. Next, they were asked to provide 20 proposals of distribution amount as proposer for a latter unknown participant. The participants were told that one trial would be randomly chosen through all the tasks, and they would be paid according to its allocation. Therefore, they received no feedback from a responder. We defined “60:40” and “50:50” as “*fair offer*”, and “90:10”, “80:20” and “70:30” as “*unfair offer*” based on a previous study [17].

### Analysis of UG data

We compared the acceptance rates of “*fair offer*” and “*unfair offer*” in terms of a responder. The smallest acceptable offered amount of each participant was also compared between groups. The mean distribution amount of proposed offers was adopted as a proposer index.

### Post-experimental questionnaires for strategy

Post-experimental questionnaires to confirm comprehension and strategy of the experiments were performed. In the questionnaires, the decision strategies selected according to findings of previous studies were queried using a 7-point Likert scale (1: “very low” - 7: “very high”) in the following six items: fairness, hostility, reputation, high-mindedness, disregard aversion, and monetary gain [18–21]. Comparing the distribution of the strategy in individuals, subtraction of the mean score of the six items from each raw score was separately calculated for all three roles in order to attenuate the variance of the scoring method. Between-group differences were examined using two-tailed independent sample t-tests (*p* < 0.05). Because we selected these items on the basis of the hypotheses, we did not perform any correction for multiple comparisons. Correlation analyses between the offered amount and each strategy were also examined using Pearson’s correlation coefficients with significance defined as *p* < 0.05. The main purpose of these correlation analyses was to disclose that participants properly understood the task. We predicted that participants who emphasize monetary gain would keep higher amounts for themselves, and that those who emphasize fairness would allocate the amount more fairly.

## Results

### Demographics

There was no significant difference in IQ between the HC and AN groups (Table 1). Body mass index (BMI) was much lower (*p* < 0.001) and the global score and subscales of the EDE-Q [22] were significantly higher in the AN group.

**Table 1:** Comparisons of the demographics and clinical characteristics in patients with anorexia nervosa and healthy controls

	Mean (SD, range) or Number			*p* value
	HC	AN		
participants	22	24		
		ANR:12	ANBP:12	
age	34.59 (9.7, 20–54)	35.9 (10.0, 20–56)		.65
BMI (kg/m^2^)	21.8 (3.4, 16.5–28.2)	14.3 (2.7, 10.1–20.7)		< .001
		ANR: 12.4 (1.7, 10.1–13.8)	ANBP: 16.1 (2.3, 13.7–20.7)	
JART	105.3 (8.1)	102.2 (9.1)		.31
EDE-Q				
Global score	0.7 (0.6)	2.2 (1.8)		< .001
Restraint	0.4 (0.7)	1.7 (1.9)		.006
Eating Concern	0.1 (0.1)	2.0 (1.9)		< .001
Shape Concern	1.3 (1.0)	2.9 (1.9)		.001
Weight Concern	1.0 (0.9)	2.3 (1.8)		.003
Duration of Illness (years)		16.7 (9.3, 5–41)		
		ANR: 11.4 (6.5, 5–30)	ANBP: 22.0 (8.6, 6–41)	

Two-tailed *p*-values of two-sample t-test were calculated in all analyses

*SD* standard deviation, *HC* Healthy Control, *AN* Anorexia Nervosa, *ANR* Anorexia Nervosa Restricting Type, *ANBP* Anorexia Nervosa Binge-Eating/Purging Type, *BMI* Body Mass Index, *JART* Japanese Version of the National Adult Reading Test short form, *EDE-Q* Eating Disorder Examination-questionnaire 6.0

### Task performance

**Fig. 1:**
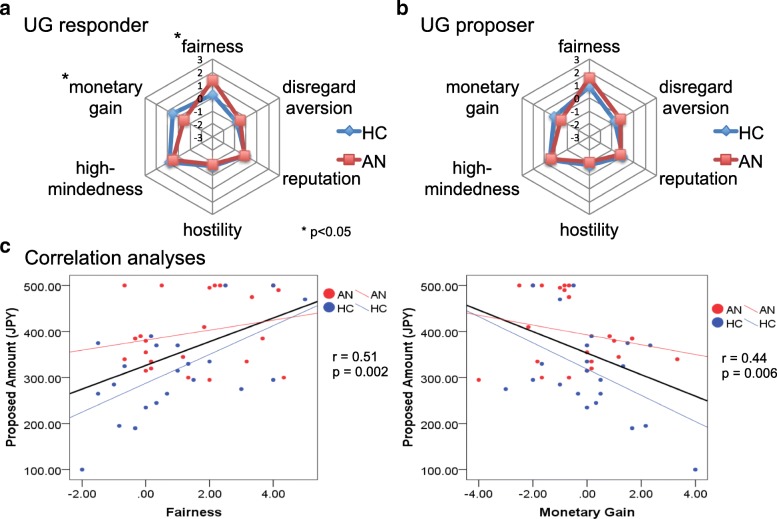
Distribution strategy in the UG of AN and HC. **a** The decision strategies of the responder of UG are depicted. The AN group placed more emphasis on ‘fairness’ and less on ‘monetary gain’ compared with the HC group. **b** The decision strategies of the proposer of UG are depicted. No significant difference was detected. **c** Correlation analyses of the offered amount by the proposer with preference for ‘fairness’ (left) and that for ‘monetary gain’ (right). Both of the Pearson’s r scores (solid black lines) are the correlation coefficients of the combined groups (HC and AN). The mean offered amount was correlated positively with ‘fairness’ and negatively with ‘monetary gain’ for all participants

Participants were asked about their understanding of the task using a 7-point Likert scale (1: “Not at all” - 7: “Completely”). There was no significant difference between the 2 groups, indicating a similar understanding of tasks (HC mean: 6.77, AN mean: 6.44). No significant difference was detected in the acceptance rates of ‘fair offer’, and the AN group had a tendency to reject ‘unfair offer’ more frequently than HC (Table 2). The smallest acceptable offered amount by AN was significantly higher than that by HC. As proposer, the AN group offered a significantly higher amount to the opponent than HC. Decision-making strategies of the two roles are shown in Fig. 1a and b. When playing the role of responder, the AN group put more emphasis on fairness, and in contrast, less on monetary gain compared with the HC group (*p* = 0.046, *p* = 0.042, respectively) (Fig. 1a, b). The mean offered amount of the HC group showed positive correlation with emphasis on ‘fairness’ (*r* = 0.61, *p* = 0.003) and negative correlation with emphasis on ‘monetary gain’ (*r* = 0.47, *p* = 0.028), and that of the AN group showed no significant correlation with either item (‘fairness’: *r* = 0.31, *p* = 0.14; ‘monetary gain’: *r* = 0.33, *p* = 0.12) (Fig. 1c). The results of correlation analyses in the HC group were consistent with our assumption. We could not find any significant difference between the correlation coefficient of HC and that of AN, neither in ‘fairness’ nor in ‘monetary gain’ (‘fairness’: *p* = 0.22, ‘monetary gain’: *p* = 0.60).

**Table 2:** Comparison of the behavioral data of the ultimatum game in patients with anorexia nervosa and healthy controls

	Mean (SD)		*p* value
	HC	AN	
UG responder			
Acceptance rate of ‘unfair offer’ (%)	70.8 (34.6)	51.0 (41.8)	.089
Acceptance rate of ‘fair offer’ (%)	92.6 (14.2)	93.8 (16.9)	.81
Minimum acceptable amount (JPY)	159.1 (85.4)	241.7 (147.2)	.026
UG proposer			
Offered amount (JPY)	314.8 (98.6)	402.1 (79.5)	.002

Two-tailed *p*-values of two-sample t-test were calculated in all analyses

*HC* Healthy Control, *AN* Anorexia Nervosa, *UG* Ultimatum Game, *JPY* Japanese Yen

## Discussion

We detected significant differences in behavior and strategy between Japanese female AN and matched HC in UG. The results of UG indicate that excessive offer amount and excessive rejection rate reflect guilt and envy, respectively [23]. The proposer of UG has to decide the distribution, the conflict being between self-profit and preference for fairness [18, 23]. As decisive factors for the responder’s behavior, altruistic punishment and inequity aversion underlie the rejection of an unfair offer [24]. The results of the AN group indicate that the higher distribution amount as proposer and the lower accepting behavior as responder may reflect a strong preference for fairness. The decision-making strategy of responders also showed that AN patients placed great emphasis on fairness.

One explanation for the strong preference of the AN patients for fairness might be linked to the social emotions guilt and envy. In UG, individuals with more guilt were found to propose fair allocation, and those with more envy tended to reject unfair offer [25]. Eating disorder patients are believed to demonstrate high sensitivity to guilt and envy, which are not limited to eating settings [4, 5]. Therefore, exaggerated guilt and envy of AN patients can affect not only their own behavior concerning eating attitude and body shape, but also decision-making in interpersonal situations.

Another explanation for the strong preference for fairness is the fact that AN patients show psychological inflexibility motivated by demand of certainty and fear of social rejection and negative evaluation by others [26]. This inflexibility leads to over-reliance on social norms and rules for behavior to reduce ambiguity/risk and mistakes. In fact, the risk-aversive trait was detected in a decision-making study of AN [27]. AN patients may have chosen ‘excessive’ fair allocation as a more certain condition in UG in order to avoid blame. AN patients may stick to the normative principle that people ‘should’ follow and consequently make inflexible decisions in interpersonal situations. This notion might partly explain the result that, despite the exaggerated emphasis on fairness, it was not positively correlated with the mean offered amount by the proposer in AN, whereas HC showed a rational positive correlation between them.

Several limitations of the current study need to be considered. First, as the number of participants was limited, we must exercise caution regarding interpretation of the results until similar behavioral experiments can be performed with larger samples of AN patients. Second, for a better understanding of the decision-making process linked to the psychopathology of AN, the development of decision-making experiments based on other than monetary rewards is strongly awaited. Third, we selected the items of post-experimental questionnaires with strong hypothesis on the basis of previous studies and the results were reasonable and in agreement with our hypothesis. However, unfortunately, we did not perform correction for multiple comparisons. Future replication studies are recommended.

Given that poor decisions are related to poor real-life outcomes, intervention targeting impaired decision-making may improve clinical outcomes and quality of life [28]. For instance, neuroimaging studies have demonstrated the neural correlates of guilt and envy [e.g., medial prefrontal cortex [29, 30] and anterior cingulate [31], respectively], and malfunctions of these midline structures were repeatedly reported in AN [32]. Further investigations of economic behavioral experiments concurrent with neuroimaging are urgently required. Moreover, neuromodulation targeting these emotions also merits further investigation. In conclusion, behavioral economics tools are useful for the evaluation of the altered emotions and decision-making that are rooted deep in the psychopathology of AN.

## Abbreviations

AN: Anorexia nervosa
ANBP: Anorexia nervosa, binge-eating/purging type
ANR: Anorexia nervosa, restricting type
BMI: Body mass index
EDE-Q: Eating Disorder Examination-questionnaire 6.0
HC: Healthy controls
SCID: Structural clinical interview for DSM-IV Axis I Disorders
UG: Ultimatum game
